# Effectiveness and Mechanism of Resibufogenin on Human Renal Cancer Cell Caki-1

**DOI:** 10.3390/biology13121064

**Published:** 2024-12-19

**Authors:** Yuqi Wu, Yue Yang, Run Huang, Tao Li, Chunlei Wan, Lei Zhang

**Affiliations:** School of Life Science and Technology, Mudanjiang Normal University, Mudanjiang 157011, China; swxkeyan@126.com (Y.W.); cutechunlei@163.com (C.W.)

**Keywords:** Resibufogenin, renal cell carcinoma, network pharmacology

## Abstract

In this study, we investigated the effect and mechanism of Resibufogenin on renal cell carcinoma based on network pharmacology, molecular docking, and in vitro experiments. The results showed that there were 35 cross-targets between Resibufogenin and renal cell carcinoma. GO and KEGG pathway analyses indicated that Resibufogenin inhibited renal cancer cells through the vascular smooth muscle contraction signalling pathway and EGFR tyrosine kinase inhibitor resistance signaling pathway, and MAPK1, PRKCB, and Resibufogenin had strong associative activities. After different concentrations of Resibufogenin were applied to human renal cancer cells, it was found that the IC50 value was 408.2 nM, 10 nM resibufogenin could significantly inhibit cell migration (*p* < 0.0001), the percentage of apoptosis and necrosis increased dose-dependently, and the expression of genes of MAPK1 and PRKCB in the cells was significantly reduced (*p* < 0.001) in a dose-dependent manner. The above results indicate that Resibufogenin can inhibit human renal cell carcinoma through multi-targets and multi-methods, which provides a theoretical basis for the application of Resibufogenin in the treatment of renal cell carcinoma and the development of novel drugs in the future.

## Simple Summary

Renal cell carcinoma is one of the common tumors in the urinary system originating from the tubular epithelium of the kidney. The preferred treatment for renal cell carcinoma in clinical practice is still surgical treatment, which involves subtractive nephrectomy of the kidney. In addition to conventional surgical treatments, drug-targeted therapies are also commonly used, but the price of targeted drugs is expensive and also has certain side effects. Our study was conducted to find a new safe and effective drug. Resibufogenin has been proven to be an effective substance for the anti-tumor effect of huachunin. Resibufogenin is a multi-target, multi-functional, and multi-pathway inhibitor of renal cell carcinoma, and can alter the morphology of Caki-1 cells and inhibit cell proliferation. Our results provide a theoretical basis for developing novel drugs for renal cell carcinoma in the future.

## 1. Introduction

Renal cell carcinoma is one of the common tumours in the urinary system originating from the renal tubular epithelium. The preferred treatment for renal cell carcinoma in clinical practice is still surgical treatment. Patients undergo tumour-reducing nephrectomy, and in addition to conventional surgical treatment, targeted drug therapy is also commonly used. Targeted drugs can prolong the overall survival time of patients and reduce the probability of tumour metastasis to a certain extent. However, the expensive price of targeted drugs also has certain side effects, such as hypertension, hand foot syndrome, hypothyroidism, etc., which limit the therapeutic efficacy of targeted drugs. Therefore, it is urgent to find a new safe and effective drug.

**Figure 1:**
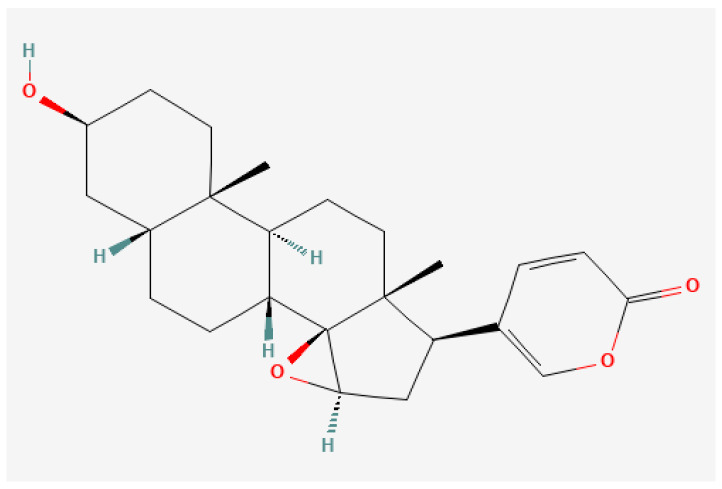
Chemical structure of the Resibufogenin.

Cinobufagin has a high medicinal value, and Resibufogenin is one of the active ingredients of toadflax and has also been proven to be the effective substance for the anti-tumour effect of huazhuxin [1]. Cinobufagin is a form of traditional Chinese medicine that is extracted from the postauricular glands and skin secretions of Bufo gargarizans Cantor or Bufo melanostictus Schneider [2]. This medication has multiple roles, such as detoxifying, alleviating pain, reviving, and rejuvenating. It is extensively utilized in treating ailments like carbuncles, gangrene, sore throats, sunstroke, abdominal pain, and vomiting [3,4]. Resibufogenin (RBG), a key bioactive compound in the bufadienolide category, is primarily obtained from toad venom. In traditional Chinese medicine, this compound has been employed for centuries in the treatment of malignant diseases and constitutes a vital element of the “cinobufacini injection”, a clinical therapeutic agent utilized in the management of advanced tumours in China. This compound has a long history in traditional Chinese medicine for treating malignant conditions and is an essential element of the “cinobufacini injection”, used clinically for advanced tumours in China [5,6]. The molecular formula of Resibufogenin is C_24_H_32_O_4_ with a relative molecular mass of 384.5 (Figure 1).

Resibufogenin can play an anti-tumour role by inducing apoptosis of tumour cells, blocking the cell cycle, and hindering cell migration and invasion. Resibufogenin can induce apoptosis in many kinds of tumour cells: Resibufogenin can act on human hepatocellular carcinoma cells Bel-7402, resulting in the increase of Bax protein expression, which has a promotion effect on the generation of apoptosis and has a synergistic effect on the decrease of Bcl-2 protein expression, and can also cause the decrease of mitochondrial transmembrane potential, the increased release of cytochrome C, the activation of caspase-3, and the inhibition of the expression of Bcl-2 protein, inducing cell apoptosis. Resibufogenin can regulate the PI3K/Akt/CREB pathway, increase the expression of caspase-3 protein, reduce the level of proto-oncogene PIM1 to inhibit the process of glycolysis, and induce apoptosis of ovarian cancer cells; Resibufogenin can regulate the PI3K/Akt/CREB pathway, increase the expression of caspase-8 protein, regulate PI3K/Akt/Akt/CREB pathway, and inhibit the process of glycolysis. PI3K/Akt/GSK3β signalling pathway induced apoptosis in gastric cancer cells MGC-803 [7,8,9]. Resibufogenin can block the cell cycle: a low dose of Resibufogenin can block the G2/M phase of gastric cancer cells.

Based on the three aspects of network pharmacology, molecular docking, and cell experiments, this study verified the inhibitory effect of Resibufogenin on renal cell carcinoma from the acquisition of the intersection target of Resibufogenin and renal cell carcinoma, the analysis of biological functions, the analysis of related signalling pathways, the strength of molecular binding ability, as well as the cellular morphology observation of Resibufogenin on renal cell carcinoma cells, such as the expression of the core target of cell proliferation, apoptosis, cell migration, and so on. The inhibitory effect of Resibufogenin on renal cell carcinoma has been verified from various perspectives, which will provide a certain theoretical basis for the research and development of novel drugs for renal cell carcinoma in the future.

## 2. Materials and Methods

### 2.1. Experimental Materials

#### Drugs, Reagents, and Cells

Resibufogenin (No. B21666) was purchased from Baijin Biotechnology Co. Ltd. (Changchun, China). FBS was purchased from Hyclone (Logan, UT, USA), Trypsin was purchased from GIBCO (San Diego, CA, USA), and Annexin V-FITC Apoptosis Detection Kit was purchased from Biyuntian (Shanghai, China). Human renal cell carcinoma cells Caki-1 were kindly donated by the Institute of Biophysics, Chinese Academy of Sciences. Websites related to network pharmacology experiments in Table 1.

Important Instruments. Inverted phase contrast microscope (CX31) was purchased from Olympus (Tokyo, Japan), enzyme labeller (Synergy HTX) was purchased from BioTek (Winooski, VT, USA), centrifuge (2–16 KL) was purchased from Sigma (St. Louis, MO, USA), and fluorescent quantitative PCR system (StepOnePlus™) was purchased from Thermo Fisher Scientific (Waltham, MA, USA).

### 2.2. Experimental Methodology Randomized Controlled Trial

#### 2.2.1. Acquisition of Intersection Targets of Renal Cell Carcinoma and Resibufogenin

Gene Expression Omnibus (GEO) was searched for the keyword “renal cell carcinoma”, and the dataset with high relevance was selected GSE66272 [10,11]. Then the dataset was subjected to GEO2R analysis, and *p* < 0.01, abs log FC > 2 as the screening conditions; the result was the differentially expressed gene. *p* < 0.01, abs log_2_ FC > 2 were used as the screening conditions, and the result was the differentially expressed gene of renal cell carcinoma [12].

Using PubChem, the SMILES symbols of the Resibufogenin were obtained and entered into the SwissTargetPrediction database to obtain human targets associated with Resibufogenin [13], and the targets were entered into Venny 2.1.0 to obtain the intersecting targets of renal cell carcinoma with Resibufogenin.

#### 2.2.2. Construction of Protein-Protein Interaction Networks

The intersection targets of renal cell carcinoma and ester toad toxin ligands were inputted into the STRING database to select the minimum threshold of 0.400, constructed Protein-Protein Interaction Networks (PPIs), exported to Cytoscape software 3.10.1 for beautification and analysis [13,14,15].

#### 2.2.3. Acquisition of Core Targets for Renal Cell Carcinoma and Resibufogenin

Select “Apps” > “App Manager”. Search for PPI network analysis in “App Manager”. Select the top five targets by using the algorithms of degree, MCC, NNC in cytoHubba plug-in.

#### 2.2.4. GO and KEEGGG Enrichment Analysis

The intersecting targets were imported into the Metascape website for GO enrichment analysis and GO chord mapping.

The intersecting targets were entered into the KOBAS website for KEGG Sankey mapping.

#### 2.2.5. Molecular Docking

Molecular docking was performed using Resibufogenin as a small-molecule ligand, the core target shared under all three algorithms was selected as the receptor. The ligands that are not relevant to this docking were transformed to PDB format by Open Babel and PyMOL to remove the ligands that are not relevant to this docking. AutoDock Vina was chosen for molecular docking calculations, in which small molecules are searched on the protein surface to find the most stable binding site and conformation.

#### 2.2.6. MTT Method to Detect the Effect of Resibufogenin on the Growth Activity of Caki-1 Cells

Adjust the cell concentration of 4 × 10^4^ cells/mL was inoculated into 96-well plates, and four concentrations of Resibufogenin were added, an equal volume of normal medium was added to the CK group, and three replicate wells were set up in each group. After 12 h of incubation, 10 μL of MTT solution (5 mg/mL) was added to each well, and 100 μLDMSO was added to each well after 4 h. The absorbance value of each well was measured at 490 nm on an enzyme lab. The effect of Resibufogenin on the growth activity of Caki-1 cells was calculated according to the formula: cell growth activity = experimental group/control group. The IC50 of the Resibufogenin on Caki-1 cells was calculated using GraphPad Prism 8.3.0.

#### 2.2.7. Scratch Experiment Detection Caki-1 Cells in Logarithmic Growth Phase Were Parallel Scratched in 6-Well Plates

The experiment was carried out in two groups. One group was the CK group, and the other group was four configured concentrations of Resibufogenin. After 12 h of drug action, the change of scratch area after 12 h of the same position was observed under the microscope. The effect of Resibufogenin on the growth activity of Caki-1 cells was finally calculated according to the formula: migration rate = [(0 h scratch area − 24 h scratch area)/0 h scratch area] × 100%.

#### 2.2.8. FCM.Caki-1 Cells in Logarithmic Growth Phase Were Cultured in 6-Well Plates

The experiment was carried out in two groups. One group was the CK group, and the other group was the final concentration of four configured concentrations of Resibufogenin for 12 h. After 12 h, trypsin was added to digest the cells. The cells were collected by centrifugation at 1000× *g* for 5 min. The cells were resuspended at 50–100,000 and centrifuged at 1000× *g* for 5 min. A total of 10 µL of propidium iodide staining solution was added after the addition of Annexin V-FITC assayed on the machine.

#### 2.2.9. RT-PCR to Detect Changes in the Expression of the Core Target in the Cell

(1) Extraction of RNA experiments were carried out in two groups, one group for the CK group, the other group for the final concentration of four configured concentrations of Resibufogenin, the drug action of 12 h. In a six-well plate, add 200 µL of RNAiso Plus, 40 µL placed in a 4 °C centrifuge in 12,000× *g*, centrifuged added 200 µL. After centrifugation, add 200 µL of isopropanol and place it in a 4 °C centrifuge at 12,000× *g*. Discard the supernatant, add 200 µL of 75% alcohol in DEPC water to each tube, mix well, place it in a 4 °C centrifuge at 7500× *g*. Centrifuge for 5 min. After drying, add about 15–20 µL of DEPC water to dissolve the precipitate, measure the concentration and purity of the extracted RNA, and store it in a −80 °C refrigerator for spare use. (2) First-strand cDNA synthesis and gDNA removal. Add an appropriate amount of Total RNA, sequentially 5×TransScript^®^ Uni All-in-One SuperMix (Beijing Quanshijin Biotechnology Co., Ltd., Beijing, China) for qPCR for 4 µL, gDNA Remover for 1 µL, add RNase-free water to 20 µL. Mix gently, incubate at 50 °C for 5 min. Heat at 85 °C for 2 min. 5 min. Heat at 85 °C for 2 min to inactivate TransScript^®^ Uni RT/RI (Beijing, China) and gDNA Remover. (3) Quantification was performed according to the reaction system and instrument settings in the table below (Table 2 and Table 3).

(4) Data collation. The relative expression of MAPK1 and PRKCB with respect to GAPDH was obtained using GAPDH as the internal reference gene and calculated as F = 2^−∆∆Ct. All data were normalised.

(5) Primer sequences (Table 4).

#### 2.2.10. Statistical Analysis and Processing

Each group of experiments was repeated at least three times. This was analysed using SPSS 20.0. GraphPad Prism was used for graphing, and two-by-two comparisons were made using the *t*-test, with *p* < 0.05 being considered statistically different.

## 3. Experimental Results

### 3.1. Intersecting Targets of Renal Cell Carcinoma and Resibufogenin

**Figure 2:**
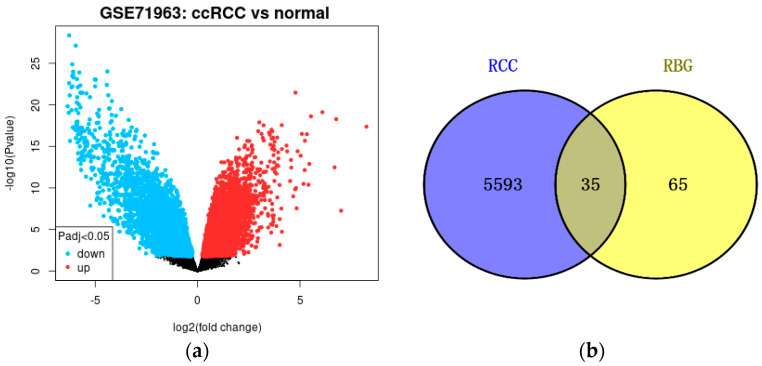
GSE66272 Volcano Map. (**a**) GSE66272 Volcano Plot (**b**) GSE66272 Volcano Plot.

The GSE66272 dataset contains 54 samples, and the dataset was analysed using GEO2R to obtain a volcano plot based on limma packages with significant differences (*p* < 0.05), where the blue dots are for proteins significantly downregulated in renal cell carcinoma group expression, and the red dots are for proteins significantly upregulated in the renal cell carcinoma group expression (Figure 2a). A total of 5628 differentially expressed genes in renal cell carcinoma were obtained based on the dataset GSE66272 in the GEO database. A total of 100 relevant targets of Resibufogenin were obtained based on SwissTargetPrediction. We obtained 35 targets of renal cell carcinoma intersecting with Resibufogenin (Figure 2b). Among the 35 targets, they include CTSS, PTGS1, PIK3CG, TTK, PYGL, etc.

### 3.2. PPI Network of Intersecting Targets of Renal Cell Carcinoma with Resibufogenin

**Figure 3:**
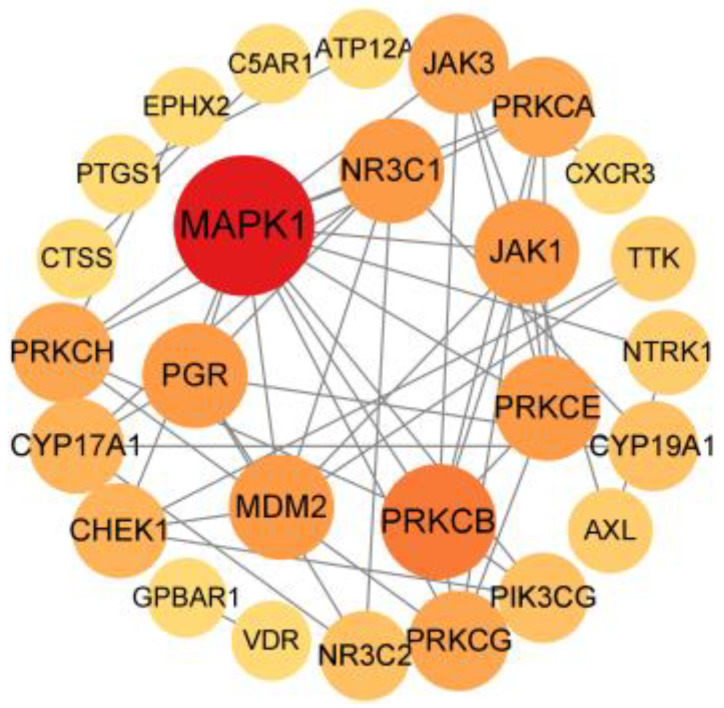
Protein-protein interaction network of renal cell carcinoma and Resibufogenin.

The intersecting targets were entered into the STRING database, the darker colour represents a higher degree value. Nodes with higher degree values were MAPK1, PRKCB, MDM2, NR3C1, PGR, JAK1, and PRKCE. Among them, the degree values of MAPK1 and PRKCB were higher than those of other targets, indicating that MAPK1 and PRKCB play a very important role in the protein interactions network of Resibufogenin action in renal cell carcinoma (Figure 3).

### 3.3. Core Targets of Renal Cell Carcinoma with Resibufogenin

**Figure 4:**
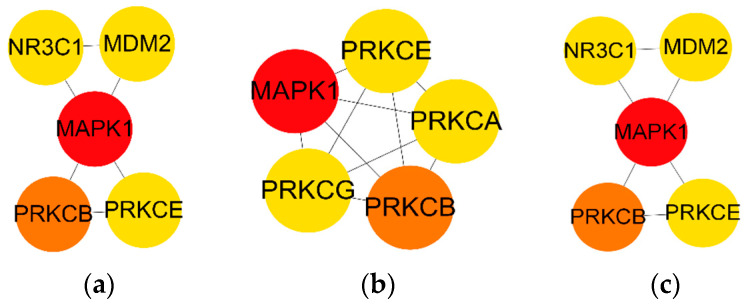
The top five ranked targets across three algorithms ((**a**). Degree; (**b**). MCC; (**c**). NNC).

The 35 intersecting targets were analysed by three algorithms, namely, degree, MCC, and NNC (Table 5). In the MCC algorithm, the top five core targets were MAPK1, PRKCA, PRKCB, PRKCE, and PRKCG (Figure 4b), and the MCC algorithm was able to make better. The top five core targets under the NNC algorithm were NR3C1, MMDM2, MAPK1, PRKCB, and PRKCE (Figure 4c). The NNC algorithm was able to make better prediction accuracy but was not sensitive to abnormal targets. MAPK1 and PRKCB were the core targets under all three algorithms.

### 3.4. GO and KEGG Enrichment of Intersecting Targets GO Enrichment Analysis

**Figure 5:**
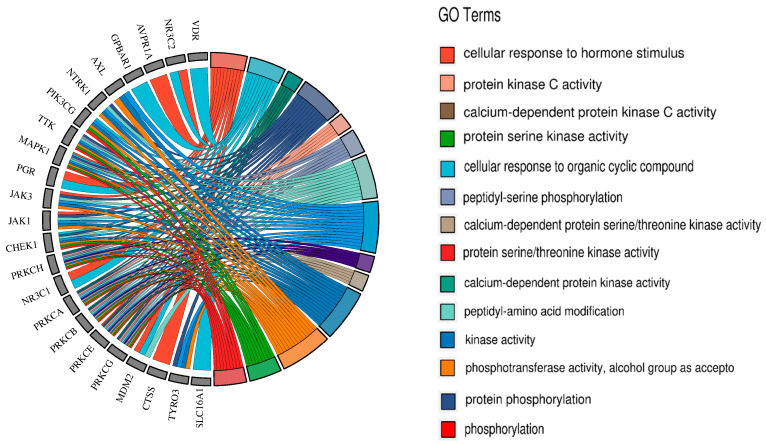
GO enrichment analysis of intersecting targets.

The results showed that the 35 targets involved 344 GO molecular functions, 2007 GO biological processes, and 204 GO cellular components. Therefore, Resibufogenin may affect renal cell carcinoma by acting on molecular functions such as the lumen of ficolin-1-rich granules, receptor complexes, and extrinsic components of membranes, as well as on biological processes such as phosphorylation and cellular activation (Figure 5).

**Figure 6:**
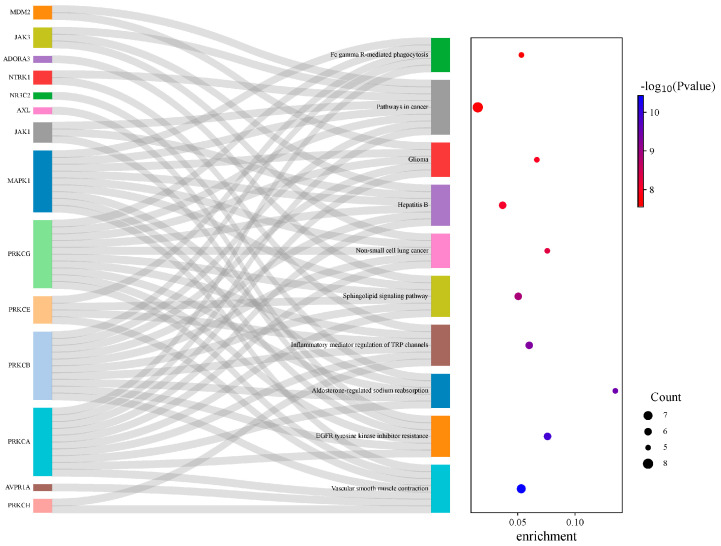
KEGG enrichment analysis of intersecting targets.

KEGG enrichment analysis showed that these 35 targets involved 164 signalling pathways, of which seven targets, PRKCH, AVPR1A, PRKCA, PRKCB, PRKCE, PRKCG, MAPK1, were able to regulate the vascular smooth muscle contraction signalling pathway, and six targets, PRKCA, PRKCB, PRKCG, MAPK1, JAK1, and AXL, were able to regulate the EGFR tyrosine kinase inhibitor resistance signalling pathway. Six targets can regulate the EGFR tyrosine kinase inhibitor resistance signalling pathway, PRKCA, MAPK1, PRKCB, PRKCG, NR3C2, five targets can regulate the aldosterone sodium reabsorption signalling pathway, and PRKCA, ADORA3, PRKCB, PRKCE, PRKCG, MAPK1, can regulate the sphingolipid signalling pathway. The multiple targets in Resibufogenin are distributed in different metabolic pathways and regulate renal cell carcinoma by regulating different signalling pathways (Figure 6).

### 3.5. Molecular Docking of the Resibufogenin Receptor with the Screened

**Figure 7:**
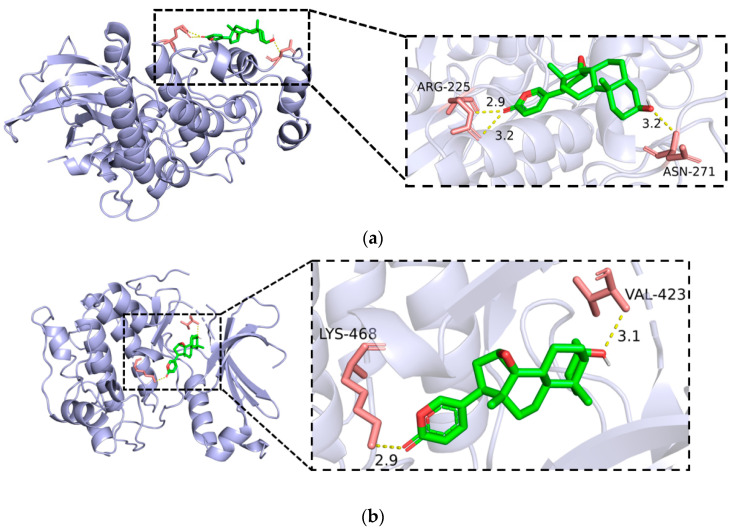
Molecular docking results ((**a**). Resibufogenin with MAPK1; (**b**). Resibufogenin with PRKCB).

MAPK1 and PRKCB, respectively, showed that Resibufogenin binds to MAPK1 with a binding energy of −7.6 kcal/mol, and Resibufogenin interacts with the amino acid residues of MAPK1, ARG-225, and ASN-271 by forming a hydrogen bond (Figure 7a). Resibufogenin binds to PRKCB with a binding energy of −9.0 kcal/mol, and Resibufogenin interacts with PRKCB amino acid residues VAL-423 and LYS-468 to form a hydrogen bond (Figure 7b).

### 3.6. Morphological Changes of Caki-1 Cells After 12 h and 24 h of Resibufogenin Under Inverted Phase Contrast Microscope

**Figure 8:**
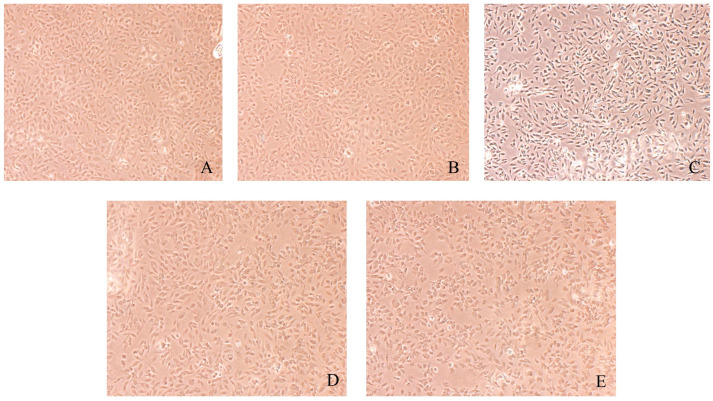
Resibufogenin drug acts on Caki-1 12 h (100×). ((**A**). CK; (**B**). 10 nmol/L; (**C**). 50 nmol/L; (**D**). 100 nmol/L; (**E**). 200 nmol/L).

**Figure 9:**
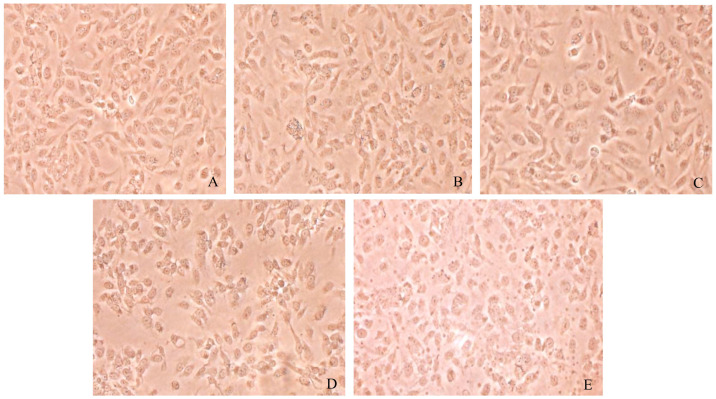
Resibufogenin drug acts on Caki-1 12 h (400×). ((**A**). CK; (**B**). 10 nmol/L; (**C**). 50 nmol/L; (**D**). 100 nmol/L; (**E**). 200 nmol/L).

**Figure 10:**
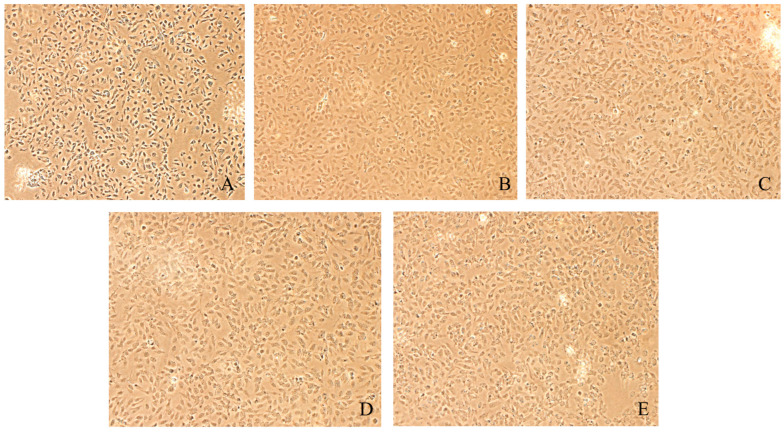
Resibufogenin drug acts on Caki-1 24 h (100×). (**A**). CK; (**B**). 10 nmol/L; (**C**). 50 nmol/L; (**D**). 100 nmol/L; (**E**). 200 nmol/L).

**Figure 11:**
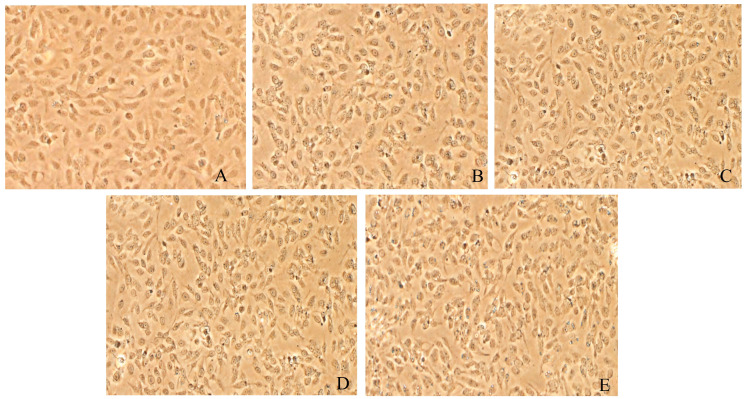
Resibufogenin drug acts on Caki-1 24 h (400×) ((**A**). CK; (**B**). 10 nmol/L; (**C**). 50 nmol/L; (**D**). 100 nmol/L; (**E**). 200 nmol/L).

**Figure 12:**
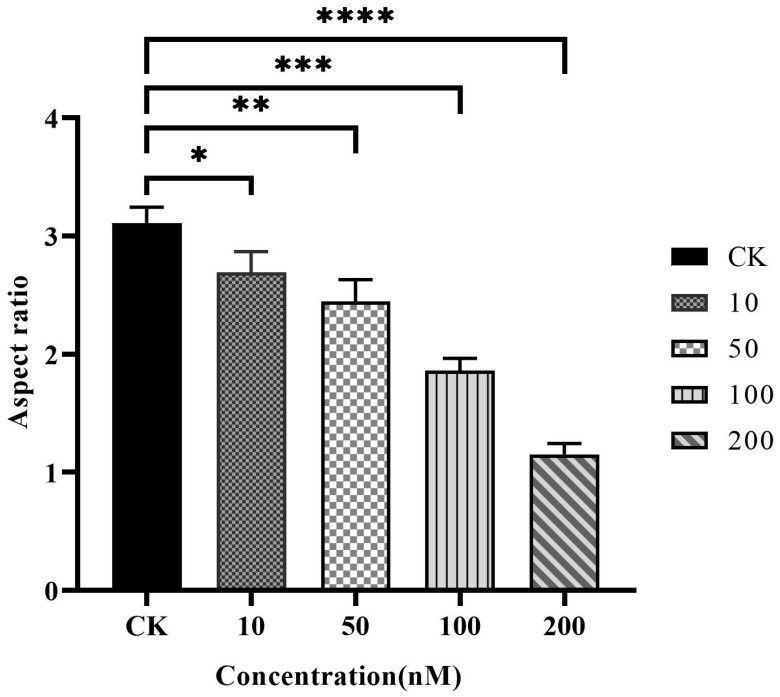
The aspect ratio on Caki-1 24 h. (* *p* < 0.05; ** *p* < 0.01; *** *p* < 0.001; **** *p* < 0.0001).

Caki-1 cells in the CK group were uniform in size, regular in morphology, with remarkable heterogeneity and adherence to the wall. After Resibufogenin was used in Caki-1 cells for 12 h and 24 h, respectively, with the increase of concentration, the cells changed from shuttle shape to round shape, the surface of the cells gradually raised, apoptotic vesicles appeared, and some of the cells appeared to have the phenomena of vacuolisation, cell fragmentation, and cytolysis. It can be seen that Resibufogenin can inhibit the proliferation of renal cell carcinoma cells and induce apoptosis to a certain extent (Figure 8, Figure 9, Figure 10 and Figure 11). In general, under normal adherent culture conditions, the aspect ratio of Caki-1 cells is roughly 1–3. Compared with normal renal carcinoma cells, the aspect ratio of different concentrations of ester bufogenin was dose-dependent, which inhibited migration and induced apoptosis (Figure 12).

### 3.7. MTT Assay to Detect the Effect of Resibufogenin on the Growth Activity of Caki-1 Cells

**Figure 13:**
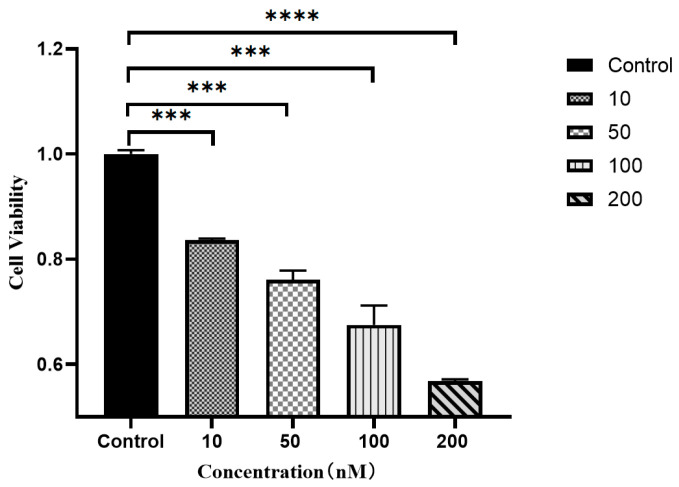
The effect of Resibufogenin on the activity of Caki-1 cells. (*** *p* < 0.001; **** *p* < 0.0001).

The results showed that after Resibufogenin was applied to Caki-1 cells in culture for 12 h, the cell activity gradually decreased with the increase of the concentration of Resibufogenin. After the four configurations of Resibufogenin were applied to Caki-1 cells in culture for 12 h, the growth activity of the cells was 83.67%, 76.12%, 67.52%, 56.85%, and 56.85%, respectively, and 76.12%, 67.52%, and 56.85%, respectively. The IC50 value of Caki-1 cells was calculated as 408.2 nM using GraphPad Prism, and the inhibitory effect of the ester toadstools on the activity of Caki-1 cells was extremely significant (*p* < 0.0001) at a concentration of 200 nmol/L (Figure 13).

### 3.8. Trace Assay to Detect the Effect of Resibufogenin on the Migration of Caki-1 Cells

**Figure 14:**
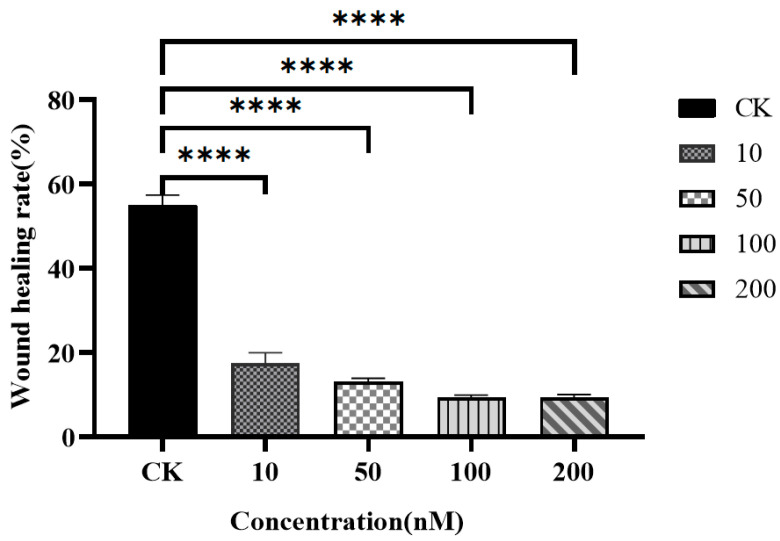
The effect of Resibufogenin on the migration rate of Caki-1 cells. (**** *p* < 0.0001).

After 12 h of incubation of Caki-1 cells, Resibufogenin had a significant inhibitory effect on the migration of Caki-1 cells at a concentration of 10 nM. As the concentration of Resibufogenin increased, the cell migration rate decreased in a dose-dependent manner (*p* < 0.0001); the type of migration analyzed is wound healing (Figure 14).

### 3.9. FCM to Detect the Effect of Resibufogenin on Apoptosis Rates in Caki-1 Cells

FCM to detect the effect of Resibufogenin on Caki-1 cells was performed using FlowJo for subsequent data analysis and visualisation. The software enables users to extract information about cell subpopulations, expression levels, and experimental results from complex FCM data. Using Annexin V and PI labelling, Q1 was defined as Annexin V-negative, PI-positive cells (necrotic cells), defining Q2 as double-positive cells (late-apoptotic cells), Q3 as Annexin V-positive, PI-negative cells (early apoptotic cells), and Q4 as double-negative cells (surviving cells).

**Figure 15:**
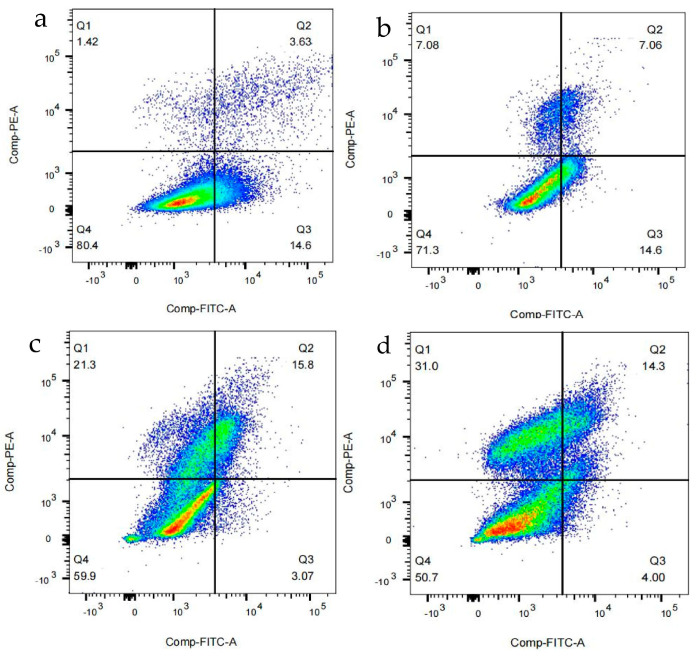
The effect of Resibufogenin on the apoptosis rate of Caki-1 cells. ((**a**). CK; (**b**). 50 nmol/L; (**c**). 100 nmol/L; (**d**). 200 nmol/L).

The results showed that the proportion of apoptotic cells and dead cells in Caki-1 cells increased with the increase of Resibufogenin, and the proportion of early apoptotic cells and dead cells in the CK group was 14.6% and 1.42%, which accounted for a total of 16.02% of the total proportion of the cells. When 200 nmol/L Resibufogenin was applied to Caki-1 cells, the proportion of early apoptotic cells and dead cells increased to 35%. The results suggest that Resibufogenin may play a role in renal cell carcinoma by inducing apoptosis and necrosis (Figure 15).

### 3.10. RT-qPCR to Detect Changes in the Expression of Core Targets in Cells

**Figure 16:**
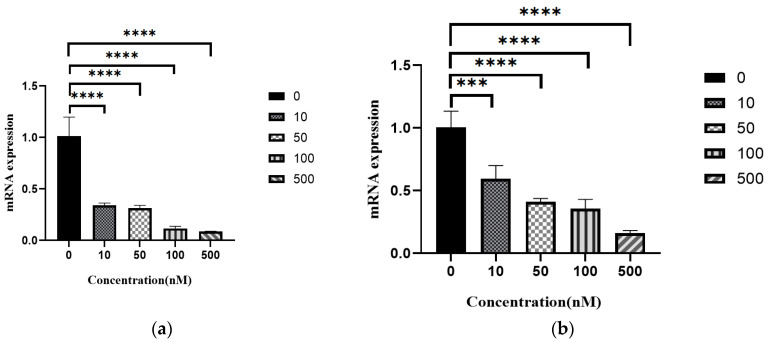
Changes in the gene expression of Caki-1 cells under the action of Resibufogenin ((**a**). MAPK1; (**b**). PRKCB). (*** *p* < 0.001; **** *p* < 0.0001).

Based on molecular docking using three algorithms to screen a total of two common targets—MAPK1 and PRKCB. 10 nM, 50 nM, 100 nM, and 500 nM of Resibufogenin in Caki-1 cells—resulted in a significant reduction in the gene expression of both MAPK1 and PRKCB (*p* < 0.001) decreased (*p* < 0.001) (Figure 16).

## 4. Discussion

As a common malignant tumour, the research and development of novel renal cell carcinoma drugs is still imminent. As an animal drug, ester bufogenin has a variety of biological activities, a strong pharmacological activity, and significant clinical efficacy. Therefore, to develop ester bufogenin as an anti-tumour drug, its components are relatively easier to be absorbed by the human body and the effect is more significant [13,14,15,16,17]. Resibufogenin is one of the active ingredients of the animal drug toadstool, and there have been various indications that Resibufogenin can exert anti-tumour effects by inducing apoptosis in tumour cells and blocking the cell cycle [13,14]. In the study of network pharmacology, we finally obtained 35 intersecting targets based on the analysis of the dataset, and the results of the PPI protein interaction network analysis of the 35 intersecting targets indicated that Resibufogenin may act on renal cell carcinoma through multiple targets. Three algorithms, namely, degree, MCC, and NNC, were used to obtain the two core targets, namely, MAPK1 and PRKCB. These two genes are involved in the processes of cell proliferation, differentiation, and apoptosis. The results of molecular docking showed that Resibufogenin could bind well with MAPK1 and PRKCB, which indicated that Resibufogenin could perform well as a small molecule drug, which is in agreement with the results of the study by El-Seedi HR, Deng Sha et al. [13,14,15,16,17].

Based on the GO enrichment analysis, we speculate that the inhibition of renal cell carcinoma by Resibufogenin may be achieved through the modulation of molecular functions such as protein kinase activity, protein tyrosine kinase activity, phosphatase binding, and phosphorylation biological processes. This also suggests that Resibufogenin can act through a variety of biological gene functions. The above corresponds to the activation and response of MAPK1 and PRKCB in the experiments, which confirms that MAPK1 and PRKCB may play important roles in the process of estero toadstool ligands in renal cell carcinoma and lays the foundation for the subsequent experiments.

The results of KEGG enrichment showed that seven targets were enriched in the PI3K-Akt signalling pathway, which is a common signalling pathway related to kidney cancer. Guo Shuangshuang et al. showed that blocking the PI3K-Akt signalling pathway and the downstream MTOR signalling pathway had an inhibitory effect on the proliferation and migration of kidney cancer cells. The studies of Zhao Lu, Cao Bo, and Li Tian showed that a number of Chinese herbal medicines could inhibit the proliferation of renal cancer cells by blocking the PI3K-Akt signalling pathway and partially led to autophagy of renal cancer cells and promoted apoptosis of renal cancer cells; and the studies of Wu Borong demonstrated that the PI3K-AKT signalling pathway was able to regulate the apoptosis of tumour cells through the regulation of a number of immune factors, as well as regulate the anti-tumour-related response [13,14,15,16,17]. Seven targets were enriched in the vascular smooth muscle contraction pathway. Studies have shown that vascular endothelial growth factor stimulates the proliferation of vascular smooth muscle cells, and angiogenesis is one of the main hallmarks of carcinogenesis.

By observing the morphological changes in the cells, it was concluded that Resibufogenin may have caused apoptosis or necrosis by disrupting the cell membrane, the cytoplasmic membrane, or the structure of the cell’s skeleton. This is similar to the results of the tumour-inhibition study conducted by Tang Xinwei et al. suggesting that Resibufogenin is able to produce morphological changes in renal cancer cells [18,19,20,21].

MTT assay showed that ester bufogenin could inhibit the proliferation of Caki-1 cells in a dose-dependent manner. YanZhou et al. showed that ester bufogenin inhibited the proliferation of myeloma cells, and XunZhang et al. showed that ester bufogenin inhibited the proliferation of glioma [18,19]. This study is similar to the results of the above studies, indicating that ester bufogenin can inhibit the proliferation of tumour cells. The experimental results also showed that ester bufogenin could achieve strong inhibitory effect at a small concentration. Cell migration is a directional movement through cell morphism, which is common in normal cells and tumour cells, and is involved in cancer metastasis, inflammatory response, and body growth and development. By studying the migration of cells through scratch experiments, we can quickly and efficiently understand the interaction of various factors such as drugs and external forces on cells, so as to explore the cancer metastasis in the body. Scratch tests showed that ester bufogenin had a significant inhibitory effect on the migration of Caki-1 cells at a low concentration, and cell mobility decreased with the increase of ester bufogenin concentration in a dose-dependent manner. This is similar to the experimental results of XunZhang et al., both of which proved that ester bufogenin has the ability to inhibit tumour cell migration at a small concentration [22].

Based on flow cytometry, the apoptosis assay was similar to the inhibitory effects of Resibufogenin on other tumour cells in previous studies, which have shown that Resibufogenin are able to inhibit the proliferation and induce apoptosis of tumour cells on a nanoscale scale, in a dose-dependent manner [2,3,4,5,6].

MAPK1 is one of the important targets in several signalling pathways such as MAP kinase signalling pathway, EMT signalling pathway, TGF-β, etc., which are closely related to cell proliferation, differentiation, and apoptosis [23,24,25,26]. The results of RT-qPCR experiments suggest that Resibufogenin may inhibit renal cell carcinoma by regulating MAPK1 in a dose-dependent manner. This regulation is dose-dependent. Various studies have shown that MAPK1 is aberrantly expressed in a variety of tumour cells such as breast, gastric, lung, colorectal, breast, and pancreatic cancers [27,28,29,30,31,32,33,34,35]. These indicate that MAPK1 is a central kinase in a variety of malignant tumours. Our study suggests Resibufogenin will have the potential to be applied as a targeted inhibitor of MAPK1 in the treatment of renal cancer, providing theoretical support for the clinical application of Resibufogenin.

Protein kinase is one of the important targets on multiple signalling pathways such as EGFR tyrosine kinase inhibitor resistance signalling pathway, MAPK signalling pathway, ErbB signalling pathway, Ras signalling pathway, Rap1 signalling pathway, and so on, which are related to tumour cell proliferation, differentiation, tumour development, as well as immunotherapy in the process of anti-tumour [33,34,35,36]. Various studies have shown that the expression of PRKCB is significantly increased in a variety of tumour cells such as lung cancer, nasopharyngeal carcinoma, human non-small cell carcinoma, and other malignant tumour cells. These studies suggest that PRKCB can be used as a potential tumour marker. Our study suggests that Resibufogenin may be used as a targeted inhibitor of PRKCB in the treatment of kidney cancer, which provides theoretical support for the clinical application of Resibufogenin [32,33,34,35].

In summary, based on the three aspects of network pharmacology, molecular docking and in vitro experiments, the present study verified the ability of Resibufogenin to inhibit renal cell carcinoma from multiple perspectives, such as the acquisition of the intersection target of Resibufogenin-renal cell carcinoma, the analysis of biological functions, the analysis of related signalling pathways, the strength of molecular binding ability, and the observation of the morphology of the cell, the proliferation of cells, the apoptosis, the migration of the cells and the expression of the related core targets of the Resibufogenin on the kidney cell carcinoma cells, Caki-1 cell [36,37,38,39,40]. It was verified that Resibufogenin could inhibit renal cell carcinoma in multiple targets, multiple functions, and multiple pathways, and could change the morphology of Caki-1 cells, inhibit cell proliferation, inhibit cell migration, induce apoptosis and necrosis, and regulate the expression of MAPK1 and PRKCB in Caki-1 cells. It provides a certain theoretical basis for the development of novel drugs for renal cell carcinoma in the future.

## 5. Conclusions

Related genes were screened through network pharmacology, and 35 targets were obtained by intersection. MAPK1 and PRKCB were the common core targets under the three algorithms. Molecular docking also verified that Resibufogenin had strong binding activity with MAPK1 and PRKC. GO enrichment and KEGG enrichment on the basis of intersecting target PPI networks further demonstrated that Resibufogenin can play a role in renal cell carcinoma through multiple targets, multiple pathways, and multiple biological functions. Cytological experiments showed that Resibufogenin was applied to Caki-1, and its cell morphology, proliferation, and migration were significantly inhibited in a dose-dependent manner. Resibufogenin can significantly induce the apoptosis and necrosis of Caki-1. Resibufogenin can affect Caki-1 by inhibiting MAPK1 and PRKCB.

## Figures and Tables

**Table 1:** Related websites.

Name	Websites
GEO database	https://www.ncbi.nlm.nih.gov/geo/ (accessed on 17 July 2024)
SwissTargetPrediction	http://swisstargetprediction.ch/ (accessed on 18 July 2024)
Venny 2.1.0	https://bioinfogp.cnb.csic.es/tools/venny/ (accessed on 18 August 2024)
STRING	https://cn.string-db.org/ (accessed on 17 August 2024)
Metascape	https://metascape.org/gp/index.html#/main/step1 (accessed on 18 August 2024)
KOBAS	http://bioinfo.org/kobas (accessed on 5 September 2024)
microfinance	https://www.bioinformatics.com.cn/ (accessed on 5 September 2024)
RCSB PDB	https://www.rcsb.org/ (accessed on 5 September 2024)
PubChem	https://pubchem.ncbi.nlm.nih.gov/ (accessed on 5 September 2024)

**Table 2:** RT-PCR reaction system.

Reagents and Components	Volume (µL)
SYBR Premix Ex Tag	10
RNase Free ddH O_2_	6.4
Forward Primer	0.8
Reverse Primer	0.8
cDNA	2
Total	20

**Table 3:** RT-PCR instrument programme settings.

Point	Temp	Times
None	95 °C	2 min
	95 °C	10 s
Quantification (40×)	60 °C	20 s
	72 °C	20 s
	95 °C	20 s
Melting Curve	65 °C	1 min
	95 °C	Continuous
None	40 °C	1 min

**Table 4:** Primer Sequences.

Gene Name	Primer Sequences (5′-3′)
MAPK1	Forward: GTGGTCGTTGAGGGCAATG
	reverse: GTGGTCGTTGAGGGCAATG
PRKCB	Forward: GTGGTCGTTGAGGGCAATG
	reverse: TGTCTCATTCCACTCAGGGTT
GAPDH	forward: CCAGGTGGTCTCCTCTGACTTC
	reverse: GTGGTCGTTGAGGGCAATG

**Table 5:** The top five ranked targets across three algorithms.

Degree	MCC	NNC
MAPK1	MAPK1	MAPK1
PRKCB	PRKCA	PRKCB
PRKCE	PRKCB	PRKCE
MDM2	PRKCE	MDM2
NR3C1	PRKCG	NR3C1

## Data Availability

Data are available upon reasonable request.
